# Risk factors of heart failure among patients with hypertension attending a tertiary hospital in Ibadan, Nigeria: The RISK-HHF case-control study

**DOI:** 10.1371/journal.pone.0245734

**Published:** 2021-01-25

**Authors:** Ayodipupo S. Oguntade, IkeOluwapo O. Ajayi

**Affiliations:** 1 Department of Medicine, University College Hospital, Ibadan, Oyo State, Nigeria; 2 Department of Epidemiology and Medical Statistics, University of Ibadan, Ibadan, Oyo State, Nigeria; Maastricht University Medical Center, NETHERLANDS

## Abstract

**Aim:**

Hypertension is the leading cause of heart failure (HF) in sub-Saharan Africa. Preventive public health approach to reduce the scourge of HF must seek to understand the risk factors of HF in at-risk populations. The aim of this study was to characterize the risk factors of HF among patients with hypertension attending a cardiology clinic.

**Methods and results:**

One hundred and one (101) case-control age- and sex-matched pairs were recruited. The study population were adults with a clinical diagnosis of hypertensive HF (cases) and individuals with systemic hypertension without HF. They were interviewed and evaluated for cardiovascular risk factors. Associations between variables were tested with chi square test, Fisher’s exact test and independent sample t test as appropriate. Logistic regression modelling was used to determine the independent risk factors of hypertensive HF (HHF) in the study population while ‘punafcc’ package in stata12 was used to calculate the population attributable fraction (PAF) of the risk factors. Suboptimal medication adherence was the strongest adverse risk factor of HHF (medium adherence aOR: 3.53, 95%CI: 1.35–9.25; low adherence aOR: 9.44, 95%CI: 3.41–26.10) with a PAF of 67% followed by dipstick proteinuria (aOR: 4.22, 95%CI: 1.62–11.02; PAF: 34%) and alcohol consumption/day per 10grams (aOR: 1.23, 95%CI: 1.02–1.49; PAF: 22%). The protective risk factors of HHF were use of calcium channel blockers (aOR 0.25, 95%CI: 0.11–0.59; PAF: 59%), then daily fruits and vegetable consumption (aOR 0.41, 95%CI: 0.17–1.01; PAF: 46%), and eGFR (aOR 0.98, 95%CI: 0.96–0.99; PAF: 5.3%).

**Conclusions:**

The risk factors of HHF are amenable to lifestyle and dietary changes. Public health interventions and preventive cardiovascular care to improve medication adherence, promote fruit and vegetable consumption and reduce alcohol consumption among patients with hypertension are recommended. Renoprotection has utility in the prevention of HF among hypertensives.

## Introduction

Hypertension is the leading risk factor for cardiovascular diseases and cardiovascular related morbidity and mortality globally and is responsible for about 7.6 million deaths every year worldwide [1]. It is the leading cause of heart failure (HF) globally and especially in sub-Sahara Africa. Hypertension is the most frequent cause of heart failure in Nigeria accounting for up to 61% in a cohort in Abuja and 75.7% in another cohort of heart failure group in Ibadan, Nigeria [2, 3]. Despite improvement in the care of patients with systemic hypertension and development of potent anti-hypertensives, heart failure incidence continues to rise even in Nigeria [2, 4]. Hypertensive heart failure (HHF) predominantly affect younger age group in African populations thus leading to loss of economic productivity and poor quality of life [5, 6].

The search for epidemiologic risk factors for heart failure in the general population has continued in recent times. Little is known till date about the unique and specific risk factors of hypertensive heart failure in Africans and Nigerians in particular. Most of the studies done in this area in Nigeria and other sub-Saharan countries have been largely descriptive. Even though, cardiovascular risk factors are largely prevalent in the adult population in Nigeria, the specific ones responsible for HHF have yet to be characterised. Preventive public health approach to reducing the scourge of HF in patients with hypertension must seek to understand the risk factors and determinants of HF in at risk populations. This is important given that HHF is a unique entity from other HF aetiologies especially in Africans. Moreover, in clinical cardiology, early recognition and attention to preventing and treating these risk factors will lead to significant reductions in HF incidence and severity. Therefore, there is a need to determine and characterise the risk factors of HF in this population.

The RISK-HHF study is a case-control study designed as an initial step in the determination and characterization of the risk factors of HHF in Nigerian-Africans. We included 101 patients with HHF and similar number of age and sex-matched hypertensive controls without HF. The specific objectives were to determine the strength of association between various modifiable cardiovascular risk factors and HHF and to ascertain the specific combination of risk factors and their population attributable fraction (PAF) responsible for the overall HF risk among Nigerian hypertensive patients that are amenable to low-cost preventive cardiovascular health.

## Materials and methods

### Participants

This study was approved by the joint University of Ibadan and University College Hospital, Ibadan Institutional Review Board (IRB) and complied with the principles outlined in the declaration of Helsinki [7]. The study also adhered to the STROBE guidelines on observational studies [8]. Written informed consent as approved by the IRB was obtained from all study participants. Participants were recruited from the cardiology clinic and medical wards of the Department of Medicine, University College Hospital, Ibadan. Details of the study methodology have been previously described and published [9]. Participants’ recruitment started in June, 2018 and the whole study lasted about 8 months. Patients aged 18 years old or above with clinical diagnosis of HF secondary to hypertension who were attending the hospital for the first time were recruited into the study as cases. Individuals diagnosed with systemic hypertension without HF who were recently referred to the cardiology clinic for continuation of care during the period of the study were recruited as controls using simple random sampling from the list in the clinic’s register. The cases and controls were matched for age (using 5-year age range) and sex in a ratio of 1:1. Participants were screened for their eligibility against a set of inclusion and exclusion criteria. Briefly, inclusion criteria for cases were clinical diagnosis of HF secondary to hypertension, mild to severe HF based on the New York Heart Association functional classification (NYHA II-IV) and first attendance at the cardiology clinic or first hospital admission while the inclusion criteria for controls were previous diagnosis of hypertension without HF and recent attendance at the cardiology clinic. Exclusion criteria for cases were heart failure diagnoses of other aetiologies, previous myocardial infarction or history of ischaemic heart disease, chronic obstructive pulmonary disease, being pregnant and consumption of ≥80g of alcohol per day for the past 5 years while exclusion criteria for controls were previous myocardial infarction or history of ischaemic heart disease, chronic obstructive pulmonary disease, being pregnant and consumption of ≥80g of alcohol per day for the past 5 years. Details of participants recruitment are shown in S1 Fig.

Clinical diagnosis of HF was based on the modified Framingham criteria and the ESC criteria [10, 11]. A patient was considered to have HHF on the basis of self-reported history of hypertension and/or the use of anti-hypertensive medications or documented blood pressure ≥ 140/90 mmHg in their clinical records on two occasions [5]. Systemic hypertension was diagnosed based on clinical records of two mean office blood pressure readings of ≥ 140/90 mmHg at 1–4 weeks interval and/or use of anti-hypertensive medications.

Sample size (N) in each group was calculated using the formula for case control study [12]: Details of the sample size calculation can be found in the S1 File. The One hundred and one (101) participants were recruited into each group.

### Procedures

A semi structured interviewer administered questionnaire was used for data collection and responses were verified from the clinical notes. The questionnaire was developed and modified from a previous study and followed the STEPS format for epidemiologic surveys [13, 14]. The questionnaire had three sections; sections A, B and C. Section A was divided into subsections on demographic data, medical history, lifestyle risk factors, symptoms and signs of HF, medications in use, examination findings and laboratory test results. Park-years of cigarette smoked was calculated for previous and current smokers. Non-smokers were deemed to have zero pack-years. Alcohol consumption in grams was estimated from the average number of drinks consumed per day among previous and current alcohol consumers while non-drinkers were deemed to consume zero grams of alcohol. Frequency of fruits and vegetable consumption in a month were estimated using a food frequency questionnaire categorized into never/rarely, occasionally, biweekly, weekly, weekly, or daily consumption. Salt consumption was also estimated in grams per day. Recommended physical exercise was defined as at least 30 minutes of moderate intensity exercise per day or 150 minutes per week.

Section B was the assessment of medication adherence. Medication adherence was assessed using the Medication Adherence Questionnaire (MAQ) of Morisky [15]. This categorized medication adherence into three categories based on a summed score; low (>2 points), medium (1–2 points) and high (0 point).

Section C contained the coding of electrocardiographic patterns according to the modified Minnesota coding system [16, 17]. The questionnaire was pre-tested before the main study among 10 cases and 10 controls. The questionnaire was also translated into the Yoruba language which is the local language spoken by most patients attending the hospital and translated back into English.

Blood pressure measurements were obtained with a mercury sphygmomanometer according to standard guidelines at each clinic visit [18]. Systolic and diastolic blood pressure were measured at Korotkoff sounds phase I and V, respectively. Two readings were taken at intervals of at least 2 minutes, and the average of the readings was recorded [19]. If there is >5 mm Hg difference between the first and second readings, additional (1 or 2) readings was obtained, and then the average of these multiple readings was recorded [20, 21]. The average of the mean recordings of two clinic visits were reported in this paper.

Subjects were weighed without shoes and in light clothing on a standard beam balance. Height was measured to the nearest centimetre using anthropometrical plane with subjects not putting on shoes or headgear [22].

Patients had full cardiovascular examination done. Patients with any of pedal oedema, abdominal distension, engorged neck veins, orthopnoea, paroxysmal nocturnal dyspnoea, basal lung crackles and rales were considered congested. Venous blood sample (20mls) was taken for serum electrolytes, serum urea, serum creatinine and fasting serum lipids from each subject along with 5mls of urine for dipstick urinalysis. Significant proteinuria was defined as more than trace proteinuria on dipstick [23]. The New York Heart Association (NYHA) functional class was assigned at recruitment in those with heart failure.

### Statistical analysis

Data were analysed using STATA version 12 (StataCorp LLC, Lakeway Drive, College Station, Texas, USA). Normality of continuous variables were determined using Shapiro-Wilk test and histogram plots. Proportions were used to summarize variables that are categorical while categorical variables were summarized as means/medians and standard deviations/interquartile range as appropriate. Cases and controls were compared using classical non-regression tests. Association of categorical variables between cases and controls were determined using chi square test while association of continuous variables between cases and controls were determined using independent sample t-test for normally distributed variables and Mann Whitney U test for non-normally distributed variables. Univariate (age- and sex adjusted) and multivariable logistic regression were used to explore the relationship between potential risk factors and HHF risk. Variables which were statistically significant in the univariate regression analyses were used in building an iterative multivariable model which was adjusted for age and sex, dietary factors, lifestyle factors, medications and biochemical variables. Interaction between variables and each of age and sex was also tested but none was statistically significant. Interaction between smoking and alcohol consumption was tested but this was also not statistically significant. The ‘punafcc’ package in stata was used to determine the population attributable fraction (PAF) of the risk factors of heart failure as post-estimation command after multivariable logistic regression. A p value < 0.05 was considered statistically significant in all analyses.

## Results

A total of one hundred and one (101) age and sex matched case control pairs were recruited into this study. Table 1 shows the baseline characteristics of the subjects. The mean age of the subjects was 62.4 years (cases) and 60.7 years (controls) with similar proportion of males and females. Individuals with HHF were more likely to have lower education attainment and more likely to be unmarried.

**Table 1:** Baseline characteristics of study participants (data are summarized as % for categorical variables, mean±SD for normally distributed continuous variables and median (IQR) for non-normally distributed continuous variables).

Variables	Cases: HHF (101)	Controls Hypertension without HF (101)	P value
**Sociodemographic characteristics**			
Age	62.4±14.3	60.7±13.0	0.36
Sex			
Male	50 (49.5)	50 (49.5)	1.00
Female	51 (50.5)	51 (50.5)	
Tribe			
Yoruba	91 (90.1)	90 (89.1)	0.51
Igbo	6 (5.9)	3 (3.0)	
Hausa	1 (1.0)	3 (3.0)	
Others	3 (3.0)	5 (4.9)	
Domicile			
Within Ibadan	85 (84.2)	84 (83.2)	0.85
Outside Ibadan	16 (15.8)	17 (16.8)	
Marital status			
Married	70 (69.3)	83 (82.2)	0.03*
Not married	31 (30.7)	18 (17.8)	
Education			
Low education (below tertiary education)	74 (73.3)	59 (58.4)	0.03*
Tertiary education	27 (26.7)	42 (41.6)	
Occupation			0.46
Employed	62 (61.4)	67 (66.3)	
Unemployed	39 (38.6)	34 (33.7)	
**Medical history**			
Diabetes	12 (11.9)	16 (15.8)	0.41
History of Kidney disease	11 (10.9)	2 (2.0)	0.02*
Obesity	16 (15.8)	22 (21.8)	0.28
Hypertension in first degree relative	30 (29.7)	36 (35.6)	0.37
Diabetes in first degree relative	4 (4.0)	10 (9.9)	0.10
**Lifestyle risk factors**			
Exercise			
None	58 (57.4)	53 (52.5)	0.64
Below recommended level	37 (36.6)	39 (38.6)	
Recommended level	6 (5.9)	9 (8.9)	
Smoking			
Never	86 (85.1)	91 (90.1)	0.28
Previous	15 (14.9)	10 (9.9)	
Current	0 (0.0)	0 (0.0)	
Park-years of cigarette consumed	1.2 (0.5, 4.8)	0 (0, 0)	<0.001
Alcohol			<0.001**
Never	54 (53.5)	77 (76.2)	
Previous	42 (41.6)	15 (14.9)	
Current	5 (4.9)	9 (8.9)	
Alcohol 0-20g/day	69 (68.3)	82 (81.2)	0.03*
Alcohol >20g/day	32 (31.7)	19 (18.8)	
Duration of alcohol consumption (years)	12 (7, 24)	20 (10, 30)	0.18
Fruits and vegetables intake			
Rarely	18 (17.8)	17 (16.8)	<0.01*
Occasionally	62 (61.4)	43 (42.6)	
Daily	21 (20.8)	41 (40.6)	
Adding salt to food on the table			
Never/Rarely	87 (86.1)	93 (92.1)	0.36
Occasionally	10 (9.9)	5 (4.9)	
Often	4 (4.0)	3 (3.0)	
Salt intake per day (grams)	5.9 (5.9, 14.7)	5.9 (2.9, 11.8)	0.15
Medication adherence			
High	20 (19.8)	61 (60.4)	<0.001**
Medium	28 (27.7)	22 (21.8)	
Low	53 (52.5)	18 (17.8)	
**Anti-hypertensive Medications**			
β-Blockers	21 (20.8)	30 (29.7)	0.14
ARB/ACE-I	54 (53.5)	74 (73.3)	<0.01*
Calcium channel blocker	22 (21.8)	61 (60.4)	<0.001**
Diuretics	39 (38.6)	53 (52.5)	<0.05*
**Clinical profile**			
BMI (Kg/m^2^)	27.6±9.4	27.8±6.7	0.83
Waist hip ratio	0.96±0.10	0.95±0.11	0.55
Pulse (/min)	87.9±1.5	89.3±6.4	0.84
Respiratory rate (/min)	20.6±7.4	17.8±3.9	0.001**
SBP (mmHg)	126.8±23.6	145.7±20.1	<0.001**
DBP (mmHg)	79.4±18.3	86.0±19.0	0.01*
**Laboratory tests**			
Proteinuria (more than trace) by dipstick	45 (45.0)	12 (11.9)	<0.001**
Serum Urea (mg/dl)	34 (26, 50)	27 (20, 34)	<0.001**
Serum creatinine	1.1 (0.9, 1.3)	0.9 (0.8, 1.2)	<0.001**
eGFR	72.5 (24.1)	90.0 (33.4)	<0.001**

ARB: angiotensin receptor blocker; ACE-I: angiotensin converting enzyme inhibitor; BMI: body mass index; SBP: systolic blood pressure; DBP: diastolic blood pressure

*p<0.05

**p<0.001.

There was no difference in obesity, diabetes and family history of hypertension or family history of diabetes between the two groups. Individuals with HHF were more likely to have background history of kidney disease. They were also more likely to smoke more packyears of cigarette. There were more current alcohol consumers among HHF than hypertension controls but more previous alcohol consumers among HHF than hypertension controls. HHF consumed more than 20g/day of alcohol compared to hypertension controls. Hypertension controls were more likely to consume fruits and vegetables daily compared to individuals with HHF. There was no difference in salt consumption and adding salt on the table between the two groups.

Medication adherence was significantly higher among the controls than the HHF cases with about two-thirds of the controls having high medication adherence compared to one-fifth of the HHF cases. The controls were more likely to have been on ACE-Is/ARBs, calcium channel blockers and diuretics than individuals who developed HHF.

HHF cases had higher respiratory rates than controls, but lower systolic and diastolic blood pressures than the controls. Though, there was no significant difference in pulse rate between the two groups. Significant proteinuria by dipstick (more than trace proteinuria) was observed in almost half of the HHF group but only in about one-tenth of the controls. Individuals with HHF also had significantly higher serum urea and creatinine and significantly lower estimated glomerular filtration rate (eGFR).

Table 2 shows the univariate logistic regression of the potential risk factors of HHF with crude, and age- and sex adjusted odd ratios. Lower education was associated with a 1.95-fold increased risk of HHF which was only slightly attenuated by 5% in age and sex adjusted regression. History of background kidney disease was associated with a 6-fold increased risk of HHF which was attenuated by 17% in age and sex adjusted analysis. Previous alcohol consumption was associated with about 4-fold increased risk of HHF which was magnified to 6-fold increased risk of HHF in age and sex adjusted analysis. Alcohol consumption was associated with 10% increased risk of HHF (crude estimate) and 13% increased risk (age and sex adjusted) per 10g or glass of alcohol consumed per day. Daily fruits and vegetables consumption were associated with 62% reduced risk of HHF which remained almost the same in age and sex adjusted analysis. Moderate medication adherence was associated with 3.88-fold increased risk of HHF which was magnified to 4.54-fold increased risk in age and sex adjusted analysis while low medication adherence was associated with about 9-fold increased risk of HHF (crude) which was magnified to about 12-fold in age and sex adjusted analysis. Use of ARBs/ACEI-s was associated with a 58% reduced risk of HHF (crude) and 64% reduced risk in age and sex adjusted estimates. Calcium channel blockers use was associated with 82% reduced risk of HHF (83% in age and sex adjusted estimates) while diuretics use was associated with 43% reduced risk of HHF (45% in age and sex adjusted estimates). Dipstick proteinuria was associated with 6-fold increased risk of HHF in both crude, and age- and sex adjusted estimates. A mg increase in serum urea was associated with 4% increased risk of HHF (both crude, and age- and sex adjusted estimates) while a mg increase in serum creatinine was associated with 3.5-fold increased risk of HHF (crude estimate) which was magnified to 4.25-fold increased risk after age and sex adjustment. Other details are as shown in Table 2.

**Table 2:** Univariate logistic regression of potential risk factors of HF.

Variables	Unadjusted OR (95%CI)	P value	Age and sex adjusted OR (95% CI)	P value
Age (years)	1.01 (0.99, 1.03)	0.36	1.01 (0.99, 1.03)	0.36
Male sex	1.00 (0.58, 1.74)	1.00	1.00 (0.58, 1.74)	1.00
Married	0.49 (0.25, 0.95)	0.03*	0.48 (0.24, 0.96)	0.04*
Low education (below tertiary education)	1.95 (1.08, 3.53)	0.03*	1.90 (1.04, 3.49)	0.04*
History of kidney disease	6.05 (1.31, 28.04)	0.02*	5.88 (1.25, 27.62)	0.02*
Pack-years of cigarette smoked	0.98 (0.90, 1.05)	0.55	0.97 (0.90, 1.06)	0.53
Alcohol consumption				
Never	Reference category	-	Reference category	-
Previous	3.99 (2.01, 7.92)	<0.001**	6.02 (2.69, 13.49)	<0.001**
Current	0.79 (0.25, 2.49)	0.69	1.19 (0.35, 4.01)	0.78
Alcohol consumed per day (per 10g)	1.10 (0.98, 1.23)	0.11	1.13 (0.99, 1.30)	0.06
^a^Daily fruits and vegetable intake	0.38 (0.21, 0.72)	<0.01*	0.37 (0.20, 0.70)	<0.01*
Salt intake (grams/day)	1.04 (0.99, 1.09)	0.11	1.04 (0.99, 1.10)	0.09
Medication adherence				
High medication adherence	Reference category	-	Reference category	-
Moderate medication adherence	3.88 (1.83, 8.24)	<0.001**	4.54 (2.07, 9.93)	<0.001**
Low medication adherence	8.98 (4.30, 18.74)	<0.001**	12.56 (5.58, 28.27)	<0.001**
ARB/ACE-I	0.42 (0.23, 0.76)	<0.01*	0.36 (0.19, 0.68)	0.001**
Calcium channel blocker	0.18 (0.10, 0.34)	<0.001**	0.17 (0.09, 0.32)	<0.001**
Diuretics	0.57 (0.33, 1.00)	0.05	0.55 (0.31, 0.97)	0.04*
Proteinuria (more than trace)	6.07 (2.95, 12.47)	<0.001**	6.10 (2.96, 12.59)	<0.001**
Serum urea (mg/dl)	1.04 (1.02, 1.07)	<0.001**	1.04 (1.02, 1.07)	<0.001**
Serum creatinine (mg/dl)	3.50 (1.54,7.97)	<0.01*	4.25 (1.66, 10.91)	<0.01*
eGFR (ml/min/1.73m^2^)	0.98 (0.97, 0.98)	<0.001**	0.98 (0.96, 0.99)	<0.001**

^a^Fruits and vegetable intake were dichotomised into daily vs. non-daily intake.

Table 3 shows the multivariable logistic regression model of the risk factors of HHF. In fully adjusted analyses, alcohol consumption (per 10g/day) was associated with 23% increased risk of HHF, daily fruits and vegetable consumption was associated with 59% reduced risk of HHF while moderate medication adherence was associated with a 3.5-fold increased risk of HHF and low medication adherence was associated with about 9-fold increased risk of HHF. Prior use of calcium channel blockers was associated with a 75% reduced risk of HHF. Dipstick proteinuria was associated with a 4-fold increased risk of HHF while a unit increase in eGFR was associated with 2% reduced risk of HHF. The other risk factors in the initial univariate regression were not significantly associated with HHF in adjusted analyses.

**Table 3:** Multivariable logistic regression model of independent risk factors of HF.

Variables	aOR (95%CI)	P value
Alcohol consumed per day (per 10g)	1.23 (1.02, 1.49)	0.03*
^a^Daily fruits and vegetable intake	0.41 (0.17, 1.01)	0.05
Medication adherence		
High medication adherence	Reference	-
Moderate medication adherence	3.53 (1.35, 9.25)	0.01*
Low medication adherence	9.44 (3.41, 26.10)	<0.001**
Calcium channel blocker use	0.25 (0.11, 0.59)	0.001**
Proteinuria	4.22 (1.62, 11.02)	<0.01
eGFR (ml/min/1.73m^2^)	0.98(0.96, 0.99)	<0.01*

^a^Fruits and vegetable intake were dichotomised into daily vs. non-daily intake.

Only the significant variables are shown with their odds ratio in this table. Model is adjusted for age, sex, education, marital status, kidney disease, salt intake (grams/day), cigarette pack-years, ARB/ACEI use and diuretics use.

Goodness of fit of model: Hosmer-Lemeshow chi-square = 8.99; p = 0.34.

S2 Fig shows the population attributable fraction (PAF) of the significant risk factors in the multivariable model. Medication adherence, prior use of calcium channel blocker and daily intake of fruits and vegetables accounted for the highest PAF amongst the risk factors. Medication adherence showed a PAF of 67%, followed by CCBs with PAF of 59%, then daily fruits and vegetables with PAF of 46%. Dipstick proteinuria was associated with a PAF of 34%, alcohol consumption (per 10g per day) had a PAF of 22% while eGFR showed negligible PAF of 5.3%.

## Discussion

In this study, patients with HHF (cases) were matched with patients with hypertension who were not in HF (controls) by sex and 5-year age range. Comprehensive evaluation for various lifestyle and metabolic risk factors was done. This is a unique study considering that few studies have used this method in Nigeria in characterising the risk factors of HHF. The mean age of the cases and controls in this study is 62.4±14.3 years and 60.7±13.0 respectively. This is in tandem with the findings by Akintunde et al. [24] and Mene-Afejuku et al. [25] who have reported a higher mean age of 62.1±14.2 years and 64.56±11.85 years respectively among patients with HHF. However, Ogah *et al*. [5] in the Abeokuta HF registry and Ojji *et al*. [2] in Abuja have reported a mean age of 56.6±15.3 years and 54.8±13.2 years respectively. The age of the hypertension group here is higher than that reported by most investigators in Nigeria [26, 27]. However, this is because they were matched with the cases who had a higher age. There were about equal number of both sexes in this study. The patients were selected to reduce confounding from age and sex.

The rate of smoking and alcohol consumption in this study is in keeping with other epidemiologic studies in the general population and also in HF and hypertensive patients [28]. Cigarette smoke is atherogenic, vasculotoxic and pro-inflammatory causing the release of cytokines that activate the renin-angiotensin-aldosterone pathway with resultant adverse haemodynamics and myocardial toxicity and depression [29]. Intriguingly, in our study, smoking was not a significant risk factor for HF. This may reflect the low smoking prevalence among Nigerians compared to other populations. It may also be that atherogenesis plays little role in the pathogenesis of HHF unlike ischaemic HF.

Interestingly, while previous alcohol consumption was commoner among HHF, current alcohol consumption was more common among the hypertension controls despite no significant difference in duration of alcohol consumption. This may reflect the fact that individuals with HHF are usually advised to stop alcohol consumption when they develop HF symptoms. In adjusted analysis, each 10g per day intake of alcohol was associated with a 23% increased risk of HHF regardless of whether alcohol consumption was in the past or present and irrespective of duration of alcohol consumption. Earlier studies like the INTERHEART study had suggested that moderate alcohol ingestion is cardioprotective and prevents ischaemic heart disease and consequent heart failure [30]. Also, though the relationship between alcohol and heart failure is controversial, it is known that heavy alcohol intake of more than 90g per day causes alcohol associated-cardiomyopathy with myocardial burnout [31]. Evidence for lower quantities of alcohol predisposing to heart failure is controversial [32]. However, in this study, patients with significant amount of alcohol used for the classification of alcohol heart muscle disease were excluded. Thus, it seems that any amount of alcohol is associated with increased risk of heart failure even in non-ischaemic patients with hypertension who frequently have other clustered cardiovascular risk factors. Alcohol consumption was associated with a 22% population attributable fraction of the risk of HHF per 10g or glass consumed, or better put; 22% of the proportion of HF in the hypertensives would be prevented if patients stopped alcohol consumption. A recent systematic review has also shown that no amount of alcohol is safe for health [33, 34]. The high alcohol consumption in both the cases and controls echoes the findings of Laabes *et al*. [35] in an earlier report in Nigeria among individuals with hypertension and represents an opportunity for cardiovascular preventive care, given the myocardial depressive effect of alcohol and associated increased susceptibility to arrhythmias.

The role of dietary practices deserves mention. In univariate models, daily fruits and vegetables consumption was associated with about 63% reduced risk of HHF. In adjusted multivariable analysis, though, daily fruits and vegetable consumption narrowly missed statistical significance (p = 0.05), the finding is still of clinical relevance. In the INTERHEART study [30], daily fruits and vegetable consumption was associated with a 30% relative risk reduction in acute myocardial infarction. In the DASH trial [36], a diet rich in fruits and vegetables was associated with significant reduction in blood pressure. Individuals who eat food rich in carotenoids and flavonoids are more likely to have durable blood pressure control and consequent reduced risk of HHF [37]. This is a low-cost intervention in primary care. Karppi *et al*. [38] have provided a mechanistic explanation for the value of fruits and vegetables in HHF risk. In their study, individuals with low serum β-carotene had a 2.78-fold increased risk of heart failure consistent with the antioxidant benefit of carotenoids. Salt intake and adding salt on the table was not associated with HHF risk in this study. The median salt consumption by the participants was within recommended limits. Excessive salt intake in the general population leads to expanded blood volume, increased glomerular filtration and may potentiate or accelerate hypertension in predisposed individuals. It is difficult to conclude on the role of dietary practices in HHF given the often ascertainment bias in estimating dietary intake of fruits, vegetables and salt.

In this study, less than half of the participants in each group engaged in any form of exercise. Furthermore, exercise was not associated with HHF risk contrary to reports in the literature. Sedentary lifestyle and inadequate exercise have been reported as adverse cardiovascular risk factors in the general population. Having less than 150 minutes of moderate intensity aerobic exercise per week has been reported to predispose to obesity and dyslipidemia, with resultant insulin resistance and cardiovascular deconditioning [39, 40]. Sedentary lifestyle acting in concert with other adverse lifestyle risk factors thus contribute to clinical deterioration and onset of HF [41]. Moreover, poor effort tolerance and exercise deconditioning reduces quality of life, worsens obesity, aggravates neurohumoral activation, myocardial remodeling and adversely affect cardiopulmonary oxygen consumption. It has been shown to be an independent risk factor of HF in the NHANES 1 epidemiologic follow up survey published by He *et al*. [42] Pena Sanchez *et al*. [43] and Rahman *et al*. [44] have also reported similar findings. Interestingly, neither obesity nor diabetes was associated with HHF risk in this study. The role of exercise in HHF risk in Nigerian-Africans requires further investigation.

In univariate models, low education attainment was associated with 2-fold increased HHF risk even though this became non-significant in the multivariable model. This may be because those with lower education may not fully understand the need for adherence. These population of patients, coupled with their low social and family support, are at increased of adverse outcomes like HF when they develop hypertension. Low education attainment is a surrogate for low social class in Nigeria and may lead to economic inequality and disparities in access to and provision of medical care. It also limits access to information and education about healthy lifestyle and preventive healthcare. The Heart of Soweto study has reported similar findings [6]. It is well known that low education leads to low income, poor social class, poor family support and reduced affordability of medications, poor medication adherence and vulnerability to alcohol abuse, which act in concert to put this group of individuals at higher risk of disease progression.

Among patients with hypertension in the general population, only about a third are aware of their status. Among those who are aware of their hypertensive status, only about a third are on treatment with up to 40% being on inadequate treatment and remaining uncontrolled in different populations and studies [45–49]. Drug adherence was suboptimal in the present study with only 19.8% and 60.4% of the cases and controls achieving high medication adherence since they were diagnosed with hypertension. In the multivariable model, moderate medication adherence was associated with 3.5-fold increased HHF risk while low medication adherence was associated with 9-fold increased HHF risk, with medication adherence accounting for the highest PAF of HHF (67%). Poor drug adherence or non-adherence is perhaps the most common and important risk factor of HF among patients with hypertension. In the systematic analysis by Abegaz *et al*. [50], 43 to 65.5% of patients who fail to adhere to prescribed medications are hypertensives. In this study, 45.2% of hypertensive patients were non-adherent to medications, 31.2% of those hypertensives with co-morbidities were non-adherent with 83.7% of non-adherence noticed in those with uncontrolled blood pressure. Though higher percentage of women were non-adherent to medications, the risk of non-adherence was higher in men and overall Africans and Asians were the ones who are more likely to be poorly adherent to medications with 62.5% of non-adherence noted in these racial groups [50]. Similarly, in the study by Lee *et al*. [51], 22.1% of patients at risk of HF were non-adherent to medications. These patients were more likely to be men, African-American and have shorter time to readmission for HF. Corrao *et al*. [52] also reported that those with increasing grade of adherence have reduced risk of HF. It has also been found that those who used their medications 80% of the time tend to have reduced cardiovascular events [53]. These high rates of medication poor adherence in patients with hypertension and other high-risk individuals have stimulated interest in the reasons and factors promoting poor adherence to anti-hypertensives and other cardiovascular protective medications. Aggarwal *et al*. [54] in New York, reported that among patients with poor adherence to medications use, forgetfulness, polypharmacy and being symptom free were reasons why many patients fail to adhere to their medication regimen. In the longitudinal study of hypertensives by Saguner *et al*., female sex, obesity, increased number of medications and medication non-adherence were the risk factors for hypertensive crises [55]. In this study, medication non-adherence was the most important risk factor. In our present study, low medication adherence was associated with a 9-fold increased risk of HHF. This is similar to the report by Saguner *et al*. [55] Furthermore, Adeoye *et al*. [56] have reported similar suboptimal medication adherence among Nigerians with uncontrolled hypertension. This is particularly worrisome given that these were patients attending specialist clinics. Furthermore, suboptimal medication adherence was the single most important factor that accounted for much of the attributable fraction of HHF. This an important public health concern and an avenue for intensive patient education for preventive care.

Another important finding from this study is the association between the use of medications like ACEIs/ARBs, diuretics and calcium channel blockers and HHF risk. While it may be argued that this is probably linked to their efficacy in reducing blood pressure, it is important to note that there was an intriguing inverse association between calcium channel blockers use and the risk of HHF in this study. The use of calcium channel blockers was associated with a 4-fold relative reduced risk of HHF and accounted for 59% of the PAF of HHF. At present, the reason for this finding and its significance in HF care is difficult to explain as calcium channel blockers have no mortality benefit in individuals with HF with reduced ejection fraction (HFrEF). Current hypertension guidelines recommend that Africans with hypertension (without HHF) should be placed on calcium channel blockers for hypertension control. This may have biased the reported findings in favour of their ‘apparent benefit’ in those with HHF who would be unlikely to have these medications prescribed. It is also possible that among Nigerian-Africans, calcium channel blockers achieve better blood pressure control and may thus, reduce the risk of cardiovascular decompensation that may ensue from poor BP control. A longitudinal study would be able to investigate this phenomenon better. The recent Creole study has shown the beneficial effect of calcium channel-based drug combination therapy in Nigerians with hypertension [57]. The role of calcium channel blockers in hypertensive vascular disease and HHF require further mechanistic and epidemiologic studies in Africa.

Individuals with background kidney disease were significantly more likely to have HHF in age-and sex adjusted analysis with a 6-fold increased risk. However, this was not significant in the fully adjusted model. Also, serum urea, serum creatinine, dipstick proteinuria and glomerular filtration rate showed significant association with HHF risk in age- and sex adjusted analyses. Proteinuria and glomerular filtration rate remained significant risk factors for HHF in the multivariable model, thus supporting the critical neurohormonal relationship between the heart and the kidneys in what has been termed as the ‘cardiorenal syndrome’. Renal venous congestion and endothelial activation may explain the proteinuria in HHF given that proteinuria is a marker of renal and cardiovascular disease. Furthermore, overt proteinuria has been associated with cardiac remodeling, worse LV and RV function and adverse outcomes in HF patients [58]. In the studies by Jackson *et al*. [59] and Shuvy *et al*. [60], macroalbuminuria was associated with 1.8-fold and 1.3-fold increased risk of death respectively. Niizeki *et al*. [61] have also shown that proteinuria is a powerful predictor of cardiac events in HF patients even after adjustment for other cardiovascular risk factors.

The HHF patients in this study had lower blood pressure, though higher respiratory rate which is in keeping with the chronic sympathetic drive in heart failure that results in reduced cardiovascular conditioning in the long-term. The reduced blood pressure in the later stage of heart failure is due to the loss of myocardial function and ejection fraction with failure of forward circulation. In this study, obesity was not associated with heart failure even though it was present appreciably in both the cases and controls. This is contrary to reports from the MESA [62], Framingham [63] studies and other published studies by Lam *et al*. [64] and Saguner *et al*. [55]. Thinness in heart failure has been regarded as the ‘obesity paradox’ [65]. However, it seems more likely that thinness in HF is a marker of chronic inflammation in longstanding disease such that these patients often present at the late stage of adipose tissue burn-out. Also, in the setting of HF, especially in HF with reduced ejection fraction (HFrEF), the heart cannot sustain adequate forward output and the pump failure in this condition is responsible for the low blood pressure and compensatory tachycardia and tachypnoea.

In summary, the significant adverse risk factors of HF among hypertensives in this study were alcohol consumption, suboptimal medication adherence and dipstick proteinuria while the protective risk factors were daily fruits and vegetable consumption and glomerular filtration rate.

The strengths of this study include the matching of cases with control which reduces the confounding effects of age and sex in HF risk. Furthermore, the use of a validated medication adherence questionnaire is another strength of this study, thus reducing subjectivity in the classification of the patients. Moreover, the risk factors identified in this study can be a focus for public health preventive interventions.

This study is not without limitations. First, this is a highly selected group of patients, though efforts have been made to reduce confounding. Thus, the finding is only generalisable to patients with hypertension who are at risk of HF. Also, recall bias may have affected the estimation of consumption of salt, vegetables and fruits and characterisation of the lifestyle habits. Coronary angiography was not done to completely rule out co-existing ischaemic heart disease. However, the definitions used to exclude ischaemic heart disease have been used in other studies in this population and the probability of misclassification of patients is low. Moreover, the prevalence of ischaemic heart disease in Nigeria is still low and hypertension still accounts for most of the HF in our population.

## Conclusion

In conclusion, this study has characterised and determined the risk factors of HF among patients with hypertension. These risk factors include lifestyle and dietary habits, medication adherence and renal function parameters. Health education should be in intensified in primary and specialist care settings. Setting up a medication adherence clinic may help to identify early those patients with poor adherence who may be at high risk of HF. A comprehensive HF registry should be established in all tertiary health institutions. This will help in further large-scale studies and validation of these results. Finally, a larger case-control study or even a cohort study is needed to confirm the findings of this research.

## Supporting information

S1 Fig RISK-HHF participants recruitment flow diagram. (TIF)

S2 Fig Bar graph showing the population attributable fraction of the independent risk factors of heart failure among individuals with hypertension. (TIF)

S1 File Sample size calculation. (DOCX)
