# Supplementary figures and images for: Risk factors of heart failure among patients with hypertension attending a tertiary hospital in Ibadan, Nigeria: The RISK-HHF case-control study
Source: PLoS One. 2021 Jan 25;16(1):e0245734. doi: 10.1371/journal.pone.0245734 (PMC7833138; doi:10.1371/journal.pone.0245734)

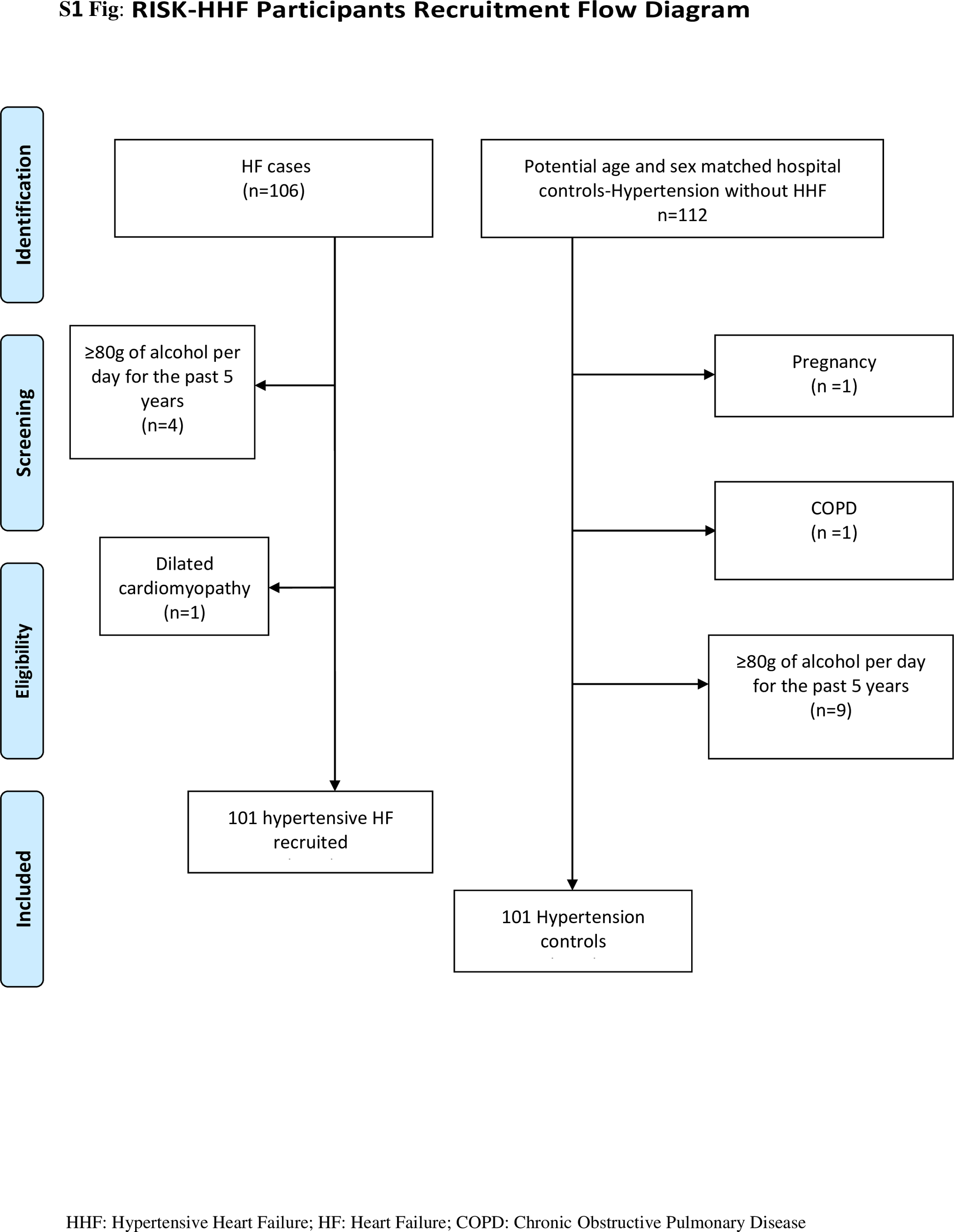

Supplement: S1 Fig — (TIF) [file pone.0245734.s001.tif]

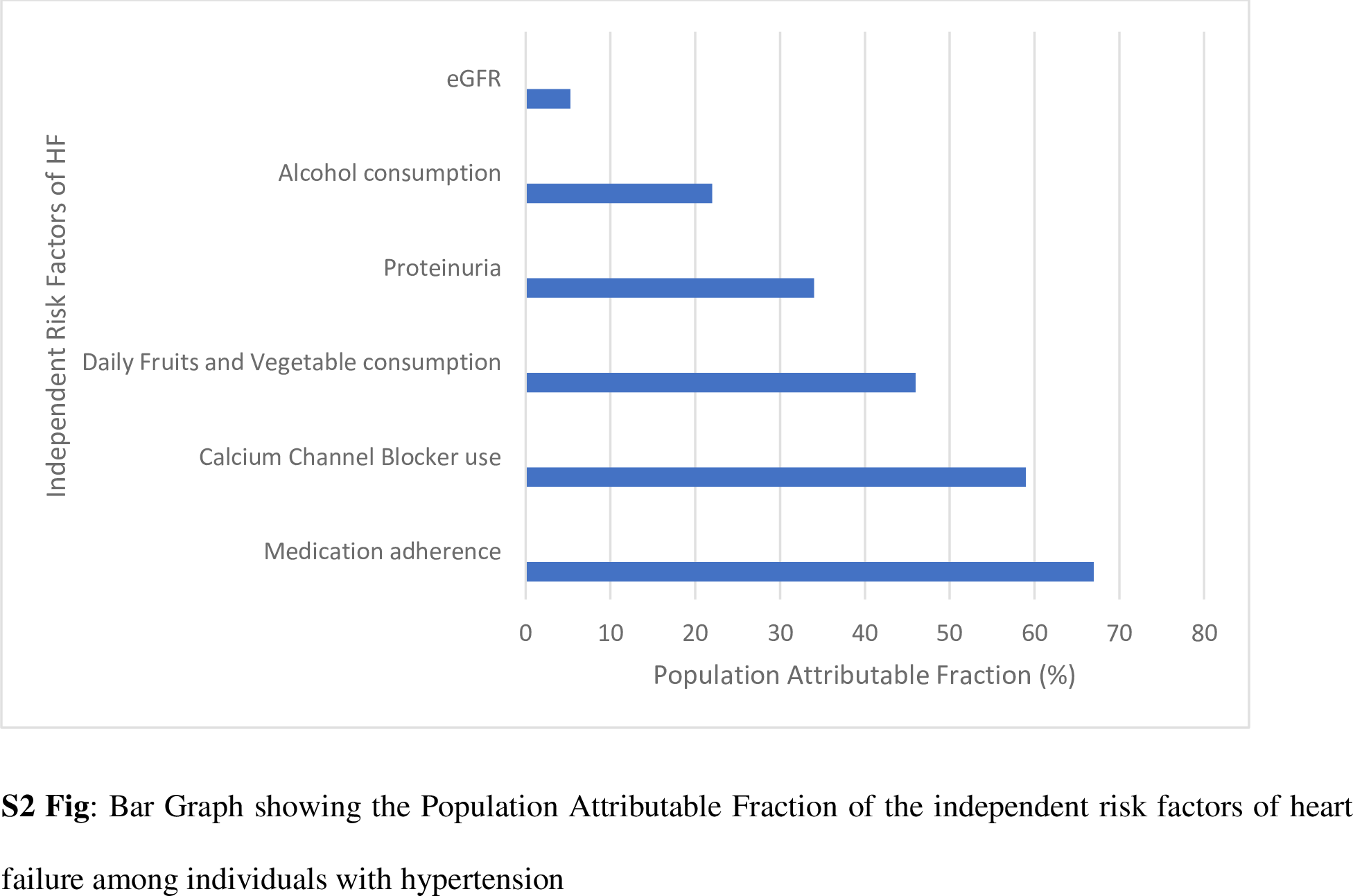

Supplement: S2 Fig — (TIF) [file pone.0245734.s002.tif]
