# Supplementary material for: Risk factors of heart failure among patients with hypertension attending a tertiary hospital in Ibadan, Nigeria: The RISK-HHF case-control study
Source: PLoS One. 2021 Jan 25;16(1):e0245734. doi: 10.1371/journal.pone.0245734 (PMC7833138; doi:10.1371/journal.pone.0245734)
Supplement: S1 File — (DOCX) [file pone.0245734.s003.docx]

**S1 File: Sample size calculation**

N= [Z_1-α/2_√2π(1-π) + Z_1-β_√π_1_(1-π_1_)+π_2_(1-π_2_)]^2^∕(π_1_-π_2_)^2^

Z_1-α/2,_ standard normal deviate at α of 0.05= 1.96

Z_1-β_ at 95% power= 1.64

π_1,_ is the prevalence of electrocardiographic LVH (dominant marker) in hypertensives in heart failure in Agomuoh and Odia^12^ =49.3%

π_2_, is the prevalence of electrocardiographic LVH in hypertensives without heart failure in Agomuoh and Odia^12^ =22%

π= (π_1_+π_2_)/2;

Thus, N= 78.5, adding a non-response rate of 25%, N becomes 98.
